# Call for Papers: PLOS Medicine Special Issue on the COVID-19 Pandemic and Global Mental Health

**DOI:** 10.1371/journal.pmed.1004010

**Published:** 2022-05-10

**Authors:** The PLOS Medicine Editors

**Affiliations:** Public Library of Science, San Francisco, California, United States of America

The editors of *PLOS Medicine* together with Guest Editors Vikram Patel, Daisy Fancourt, Toshi A Furukawa, and Lola Kola are excited to announce a forthcoming special issue devoted to the impact of the COVID-19 pandemic on global mental health.

On March 11, 2020, the World Health Organisation (WHO) declared the COVID-19 outbreak a global pandemic, just months after the first known cases were identified in December, 2019. Two years on, the world has been transformed by the SARS-CoV-2 virus: lockdowns and physical distancing have decimated social support structures for many, while loss of income, loneliness, disruptions in routine health care and reductions in physical activity have widened pre-existing health inequities. As the COVID-19 pandemic continues to evolve, a hidden pandemic of mental health disorders is already underway.

Others have already made the case for viewing the pandemic as an opportunity to reimagine global mental health and ‘build back better’. In this forward-looking Special Issue, *PLOS Medicine* and our Guest Editors wish to invite work with the potential to mitigate the mental health consequences of the current pandemic and strengthen the global response to future pandemics. Work that examines contextual differences in the mental health impacts of the pandemic and strives to elucidate underlying mechanisms is of great interest.

Areas of special interest include vulnerable populations and the pandemic’s impact on existing inequities in global mental health, health system responses to increased demand for mental health care services, evaluations of policy interventions, mental health consequences of the pandemic from a life course perspective, and public mental health. For further information, please see the full Call for Papers on our blog.

Please submit your manuscript at: http://journals.plos.org/plosmedicine/s/submit-now. The deadline is 15 July 2022.

Presubmission inquiries are not required, but do please indicate your interest in the special issue in your cover letter. Questions about the special issue can be directed to plosmedicine@plos.org.

